# Ancient Herbal Formula Mahuang Lianqiao Chixiaodou Decoction Protects Acute and Acute-on-Chronic Liver Failure via Inhibiting von Willebrand Factor Signaling

**DOI:** 10.3390/cells11213368

**Published:** 2022-10-25

**Authors:** Jiacheng Lin, Qihua Ling, Liang Yan, Bowu Chen, Fang Wang, Yihan Qian, Yueqiu Gao, Qian Wang, Hailong Wu, Xuehua Sun, Yanjun Shi, Xiaoni Kong

**Affiliations:** 1 Central Laboratory, Department of Liver Diseases, ShuGuang Hospital Affiliated to Shanghai University of Traditional Chinese Medicine, Shanghai 201203, China; 2 Department of Emergency Internal Medicine, Shuguang Hospital Affiliated to Shanghai University of Traditional Chinese Medicine, Shanghai 201203, China; 3 Department of General Practice, Shuguang Hospital Affiliated to Shanghai University of Traditional Chinese Medicine, Shanghai 201203, China; 4 Shanghai Key Laboratory of Molecular Imaging, Shanghai University of Medicine and Health Sciences, Shanghai 201318, China; 5 Abdominal Transplantation Center, General Surgery, Ruijin Hospital, School of Medicine, Shanghai Jiao Tong University, Shanghai 200025, China

**Keywords:** acute liver failure, acute-on-chronic liver failure, von Willebrand factor, traditional Chinese medicine, herbal therapy

## Abstract

Background: Acute liver failure (ALF) and acute-on-chronic liver failure (ACLF) are characterized by systemic inflammation and high mortality, but there is no effective clinical treatment. As a classic traditional Chinese medicine (TCM) formula, MaHuang-LianQiao-ChiXiaoDou decoction (MHLQD) has been used clinically for centuries to treat liver diseases. Methods: The LPS/D−GalN-induced ALF mice model and the CCl_4_+LPS/D−GalN-induced ACLF mice model were used to observe the therapeutic effects of MHLQD on mice mortality, hepatocytes death, liver injury, and immune responses. Results: MHLQD treatment significantly improved mice mortality. Liver injury and systemic and hepatic immune responses were also ameliorated after MHLQD treatment. Mechanistically, proteomic changes in MHLQD-treated liver tissues were analyzed and the result showed that the thrombogenic von Willebrand factor (VWF) was significantly inhibited in MHLQD-treated ALF and ACLF models. Histological staining and western blotting confirmed that VWF/RAP1B/ITGB3 signaling was suppressed in MHLQD-treated ALF and ACLF models. Furthermore, mice treated with the VWF inhibitor ADAMTS13 showed a reduced therapeutic effect from MHLQD treatment. Conclusions: Our study indicated that MHLQD is an effective herbal formula for the treatment of ALF and ACLF, which might be attributed to the protection of hepatocytes from death via VWF/RAP1B/ITGB3 signaling.

## 1. Introduction

Fulminant hepatic failure is a severe liver disease with a mortality rate of 30–50% [1,2] and is characterized by acute liver function damage, blood coagulation disorders, and systemic infection [3]. Fulminant hepatic failure can be divided into acute liver failure (ALF) and acute-on-chronic liver failure (ACLF) according to the presence or absence of pre-existing chronic liver disease [4,5]. ALF is primarily caused by acute viral hepatitis and acetaminophen overdose without pre-existing liver disease [6,7]. ACLF is characterized by chronic liver disease and acute hepatic decompensation. In both ACLF and ALF, hepatocyte death is the direct cause of liver failure, and consequent infection-induced inflammation is considered the frequent trigger for exacerbation [8,9]. However, to date, there is no effective treatment that can protect patients against fulminant hepatic failure. Therefore, there is an urgent need to find potentially effective treatments.

Herbal therapy originated from traditional medicine and is a valuable resource for fighting diseases. Traditional Chinese medicine (TCM) is a typical traditional medicine which uses herbs to treat diseases. Plenty of clinical experiences have been conducted in the treatment of liver injury with herbs from TCM. MaHuang-LianQiao-ChiXiaoDou decoction (MHLQD) is an ancient herb formula with over 2000 years of history. It is composed of eight herbs, including *Ephedra alata* Decne., *Forsythia giraldiana* Lingelsh., *Prunus armeniaca* L., *Phaseolus acutifolius* A.Gray, *Ziziphus jujuba* Mill., *Catalpa ovata* G.Don, *Zingiber officinale* Roscoe., and *Glycyrrhiza aspera* Pall. MHLQD was described as a formula for treating liver injury in *Treatise on Febrile Diseases Caused by Cold (Shanghan Lun)* and has been clinically used as an alternative therapy to treat jaundice, dermatosis, and nephritis [10]. MHLQD has been reported to have anti-inflammatory and anti-cell damage effects. Previous reports indicated that MHLQD alleviated CCl_4_-induced acute liver injury and α-ANIT-induced liver injury in rodents [11,12]. However, the effect of MHLQD on ACLF and ALF has not been investigated.

Von Willebrand Factor (VWF) is an oxidative stress-related factor secreted by damaged endothelial cells and megakaryocytes. Intrahepatic VWF has been considered a damaging factor that exacerbates liver injury in ACLF and ALF patients. Higher VWF in patients is associated with poor outcomes [13,14]. As an adhesion molecule, VWF recruits and activates platelets which cause the formation of microthrombus within the liver microvasculature. Ultimately, activated platelets and microthrombus lead to circulatory disorder in liver tissue circulation disorder and aggravate liver necrosis [15,16]. Taken together, the accumulation of intrahepatic VWF accelerates disease progression, and it is considered to be a novel therapeutic target for ACLF and ALF [17].

TCM formulas are composed of aqueous extracts of herbs, commonly with multiple active constituents. Mechanistic interpretation of herbal formulas has been considered difficult. In recent years, high-throughput omics techniques have been applied to comprehensively analyze the mechanism of TCM formulations [18]. Several studies have suggested that TCM formulas commonly have a major mechanism of action, which is made up of the combined effect of ingredients from TCM formulas [19,20]. Therefore, an unsupervised strategy-based method is necessary for revealing the main mechanism of a TCM formula.

In this study, we aimed to investigate the therapeutic effect and underlying mechanisms of MHLQD in the ACLF and ALF murine models. Pathological changes were observed in the liver and serum. Moreover, we explored the molecular mechanism of MHLQD via proteomics analysis and experimental verification. Results suggested that MHLQD could alleviate inflammatory injury in ACLF and ALF mice, and that the effect might be attributed to the inhibition of VWF/RAP1B/ITGB3 signaling.

## 2. Materials and Methods

### 2.1. Chemical

The following chemicals were used: LPS (L2630, Sigma-Aldrich, St. Louis, MO, USA); D−GalN (MB1931, Meilunbio, Dalian, China); CCl_4_ (C805329, Macklin, Shanghai, China); Recombinant ADAMTS13 (4245-AD, R&D systems, Minneapolis, MN, USA).

### 2.2. Animals

Male C57BL/6 mice (7–8 weeks, 20–22 g) were obtained from Shanghai JieSiJie Laboratory Animal Company (Shanghai, China). Animals were housed in the Laboratory Animal Center of Shanghai University of Traditional Chinese Medicine (Shanghai, China) at a temperature of 23 ± 3 °C with a humidity of 55 ± 5%, under a 12 h light/12 h dark cycle. All Animals were allowed ad libitum access to water and food. The animal experiments were in accordance with the institutional animal care guidelines approved by the Experimental Animal Ethical Committee of the Shanghai University of Traditional Chinese Medicine. The minimum number of animals was used.

### 2.3. Herbal Formula

The herb formula MaHuang-LianQiao-ChiXiaoDou decoction (MHLQD) comprises eight herbs, as shown in Table 1. The dosage used in the present study was calculated according to the Chinese ancient literature ‘Shanghan Lun’. The herbs were procured from the Shuguang Hospital Affiliated to the Shanghai University of Traditional Chinese Medicine. The low dose of MHLQD (40.5 g/kg) was defined according to the human-mouse body drug dose conversion coefficient, and the high dose (81 g/kg) was defined as double the low dose. The scientific names and medicinal parts of the herbs were shown in Table 1. To acquire the aqueous extracts of MHLQD, the mixtures of the collected herbs (81 g) were diluted in distilled water (600 mL) and heated at 120 °C for 1.5 h under continuous stirring. The step was repeated twice. The aqueous extractive of MHLQD was collected. To obtain the high dose of MHLQD, the aqueous extracts were concentrated at 95 °C for 2 h and then diluted to 200 mL. The high dose of MHLQD was diluted 1-fold with PBS to obtain the low dose of MHLQD.

### 2.4. Component Analysis of MHLQD by UPLC/Q-TOF-MS/MS

The aqueous extract of MHLQD was extracted with methanol (1:10, *v*/*v*) under ultrasonication (40 kHz) for 40 min and centrifuged at 12,000× *g*, 4 °C for 15 min. The supernatant was collected through a 0.22 μm filter and 4 μL of the supernatant was injected into UPLC-Q-TOF-MS/MS system for analysis. The ultra-high-performance liquid chromatography coupled with a quadrupole time-of-flight MS/MS (UPLC/Q-TOF-MS/MS) system was conducted on UPLC-Q-Orbitrap-MS (Thermo Fisher, Carlsbad, CA, USA) with an electrospray ionization source (ESI). The Waters ACQUITY UPLC HSS T3 (100 mm × 2.1 mm, 1.8 μm) (Waters, Milford, MA, USA) was used for chromatographic separation with a flow rate of 0.3 mL/min at 40 °C. The mobile phase system was as follows: Solvent A: deionized water with 0.1% formic acid (*v*/*v*); Solvent B: methanol with 0.1% formic acid (*v*/*v*). The instrumental settings of Q-TOF-MS/MS were as follows: ion source gas 1 and gas 2 were both 55 psi, curtain gas was 35 psi, ion source temperature was 100 °C, ion spray voltage floating was 3000 V in positive mode and 3200 V in negative mode, and nitrogen was used as both nebulizer and auxiliary gas. Samples were analyzed in both positive and negative ionization modes with a scanning mass-to-charge (*m*/*z*) range of 50 to 1000. Data were collected in information-dependent acquisition (IDA) mode and analyzed by Xcalibur 2.1 (Thermo Fisher, Carlsbad, CA, USA). Chemicals were identified according to chromatographic elution behaviors, mass fragment patterns, and mass spectral library Compound discover 2.1 (Thermo Fisher, Carlsbad, CA, USA).

### 2.5. Animal Model Establishment and Treatment

The animals were randomly divided into the following groups: CCl_4_ control (CCl_4_), acute-on-chronic liver failure (ACLF), low dose of MHLQD-treatment (ACLF+MHLQD Low), and high dose of MHLQD-treatment (ACLF+MHLQD High) for the ACLF model; and the normal control (NOR), acute hepatic failure (ALF), low dose of MHLQD-treatment (ALF+MHLQD Low), and high dose of MHLQD-treatment (ALF+MHLQD High) for the ALF model. In the ACLF experiments, the mice were firstly intraperitoneally injected with CCl_4_ (20 mg/kg, dissolved in olive oil) 3 times a week for 8 weeks to induce chronic hepatic fibrosis injury. On the day of the last injection with CCl_4_ (40 mg/kg), the mice were intraperitoneally injected with D−GalN (320 mg/kg) and LPS (50 ug/kg for the survival experiment and 30 ug/kg for the non-survival experiment). In the inhibitor experiments, mice were intravenously injected with 200 ug/kg (in 200 uL PBS) rADAMTS13 0.5 h after the D−GalN/LPS-treatment. After 1.5 h, the mice were intragastrically administrated with MHLQD or PBS. For the ALF experiment, the mice were intragastrically administrated with MHLQD or PBS. After 1.5 h, the mice were intraperitoneally injected with 320 mg/kg D−GalN and 30 ug/kg LPS. Then, 1.5 h after LPS+D−GalN treatment, the mice were administrated with MHLQD/PBS again. The survival rate of mice was observed during the survival experiments. For non-survival experiments, the mice were sacrificed (under anesthesia by isoflurane) 10 h after LPS+D−GalN-treatment. The MHLQD was administrated two times during an experiment and the total dose was either 40.5 g/kg (low dose) or 81 g/kg (high dose). The mice in all groups were treated with the same volume of solutions: intraperitoneal injection (0.1 mL/20 g; LPS/D−GalN or PBS), oral administration (0.2 mL/20 g; MHLQD or PBS), and tail vein injection (200 uL; rADAMTS13 or PBS; in the inhibitor experiment).

### 2.6. Short-Term Toxicity Test

The mice were randomly divided into: (1) control group (n = 10), and (2) MHLQD group (n = 10). The mice were intragastrically administrated with a high dose of MHLQD (81 g/kg) or the same volume (0.2 mL) of PBS twice daily. The body weight of the mice was measured daily. On the third day or the seventh day, the mice were sacrificed 2 h after treatment. The serum and liver tissues of mice were collected for further detection.

### 2.7. Biochemical Assay

The serum levels of ALT and AST were measured using a standard clinical automatic analyzer (Hitachi, model 7080, Tokyo, Japan).

### 2.8. ELISA

Mouse serum cytokines were detected by the IL-1β ELISA Kit (70-EK201B, MultiSciences, Hangzhou, China) and the IL-6 ELISA Kit (EMC004.96, NeoBioscience, Shenzhen, China). Blood acquired from mice was centrifuged at 3000× *g*, 4 °C, and the supernatant was purified for ELISA assay according to the manufacturer’s instructions. Optical density was measured at a wavelength of 450 nm. 

### 2.9. H&E and TUNEL Staining

The liver tissue samples were fixed in 4% neutral buffered formalin over 24 h. The fixed tissue samples were embedded in paraffin. The paraffin blocks were sliced to a thickness of 4 μm. The sections were stained with hematoxylin and eosin (H&E) (C0105S, Beyotime, Shanghai, China). Apoptotic cells were stained using the TUNEL detection kit (C1098, Beyotime, China), according to the manufacturer’s instructions. The images were recorded using an optical microscope.

### 2.10. Histochemistry and Immunofluorescence

The fixed tissue samples were embedded in paraffin. The paraffin blocks were sliced to a thickness of 4 μm. The EGTA (MVS-0098, Maxim biotechnologies, Fuzhou, China) was used for antigen repair. For histochemistry, F4/80 (28463-1-AP, Proteintech, Wuhan, China), MPO (SKU:023, Biocare Medical, Pacheco, CA, USA), VWF (A0082, DAKO, Glostrup, Denmark), and ITGB3 (MWReg30, BioLegend, San Diego, CA, USA) were used. The sections were incubated with the antibodies at 4 °C overnight. The sections were incubated with anti-rabbit IgG (#7074, Cell Signaling, Danvers, MA, USA) or anti-rat IgG (ab6734, Abcam, Cambridge, UK) for 1.5 h at room temperature and stained with DAB substrate (P0202, Beyotime, Shanghai, China). For immunofluorescence, the sections were incubated with ITGB3 (MWReg30, BioLegend, San Diego, CA, USA) overnight at 4 °C and then incubated with VWF (A0082, DAKO, Glostrup, Denmark) for 2 h at room temperature. For fluorescent staining, the sections were incubated with an Alexa 594-conjugated secondary anti-rat IgG antibody (ab150160, Abcam, Cambridge, UK) and an Alexa 488-conjugated secondary anti-rabbit IgG antibody (ab150077, Abcam, Cambridge, UK). The optical microscope and laser scanning confocal microscope were used to take images. The software ImageJ was used for quantifying the histochemistry staining.

### 2.11. Real-Time qPCR

Total RNA was extracted from the liver tissues using a Total RNA extract KIT (RP4002, Bioteke Corporation, Beijing, China), followed by reverse transcription using HiScriptII Q RT SuperMix with gDNA ripper (R222-01, Vazyme, Nanjing, China). Obtained cDNA were quantitatively detected by a PCR detection system (Quant Studio 3, Thermo Fisher, Carlsbad, CA, USA) and SYBR Green qPCR Mix (Q311-02, Vazyme, Nanjing, China). Relative gene expression was normalized with respect to β-actin. The primer sequences were as follows:

(1) IL-1β, forward: 5′-TGTGTTTTCCTCCTTGCCTCTGAT-3′,
  IL-1β, reverse: 5′-TGCTGCCTAATGTCCCCTTGAAT-3′;
(2) TNF-α, forward: 5′-TAGCCAGGAGGGAGAACAGA-3′,
  TNF-α, reverse: 5′-TTTTCTGGAGGGAGATGTGG-3′;
(3) β-actin, forward: 5′-GTGCTATGTTGCTCTAGACTTCG-3′,
  β-actin, reverse: 5′-ATGCCACAGGATTCCATACC-3′.

### 2.12. Western Blot

The protein was dissociated from liver tissues using a RIPA Lysis Buffer (89900, Thermo Fisher, Carlsbad, CA, USA) with protease inhibitor cocktails (HY-K0010, MedChemExpress, Shanghai, China). The lysate was centrifuged at 13,000× *g*, 4 °C for 15 min, and the supernatant was collected. The nuclear protein was isolated using a Nuclear and Cytoplasmic Protein Extraction Kit (P0028, Beyotime, Shanghai, China). Purified protein was mixed with a loading buffer (P0015, Beyotime, Shanghai, China) and separated on 8–12.5% gels by SDS-PAGE (P0012A, Beyotime, Shanghai, China). Isolated protein was transferred to nitrocellulose filter membranes (GSWP04700, Millipore, Billerica, MA, USA) and blocked with 5% BSA (A600903, Sangon Biotech, Shanghai, China). Membranes were incubated with primary antibodies overnight at 4 °C and then incubated with secondary antibodies for 1.5 h at room temperature. The antibodies are shown as follows: β-actin (A3854, Sigma-Aldrich, St. Louis, MO, USA); VWF (A0082, DAKO, Glostrup, Denmark); ITGB3 (MWReg30, BioLegend, San Diego, CA, USA); RAP1B (#2326, Cell Signaling, Danvers, MA, USA); NF-κB (#8242, Cell Signaling, Danvers, MA, USA); lamin B1 (#12586, Cell Signaling, Danvers, MA, USA); full-length and cleaved caspase 3 (31A1067, Novusbio, Englewood, CO, USA); kindlin-3 (#10459, Cell Signaling, Danvers, MA, USA); anti-rat IgG, Alexa 594-conjugated antibody (ab150160, Abcam, Cambridge, UK); anti-rabbit IgG, HRP-linked antibody (#7074, Cell Signaling, Danvers, MA, USA).

### 2.13. Proteomics Analysis

The protein of liver tissues was extracted by a RIPA Lysis Buffer (89900, Thermo Fisher, Waltham, MA, USA) with protease inhibitor cocktails (HY-K0010, MedChemExpress, Shanghai, China). The lysate was centrifuged at 15,000× *g* for 15 min at 4 °C. In total, 100 uL of extracted protein supernatant solution (10 ng/uL) was precipitated with 500 uL ethanol-acetone solution (1:1, *v*/*v*, containing 0.1% acetic acid) at −20 °C overnight. The protein precipitated was redissolved in 6 M guanidine hydrochloride in 50 mM NH4HCO3 solution, then incubated with DTT (2 μM) and IAA (4 μM) successively. The solution was transferred to 10 kDa cutoff filter units and centrifuged at 12,000× *g* for 40 mins at 4 °C. The concentrate was washed 3 times with 50 mM NH4HCO3 and digested for 16 h at 37 °C with trypsin, then collected by centrifugation at 12,000× *g* at 4 °C to acquire peptides. The peptides were desalted using a ZipTip C18 (Millipore, Billerica, MA, USA), and analyzed by the Q-Exective Orbitrap HF-X mass spectrometer (Thermo Fisher, Carlsbad, CA, USA). Peptides were first loaded on a trapping column and then eluted to a 75 µm analytical column at 300 nL/min; both columns were packed with Jupiter Proteo resin (3 µm, C18, 300 Å, Phenomenex, Torrance, CA, USA), and the length of the analytical column was 15 cm. MS spectra were acquired in profile mode using the Orbitrap analyzer in the *m*/*z* range between 300 and 1800 at 60,000 resolutions, and the resolution for MS/MS was set to 12,500. MS/MS spectra were searched against the UniProt Human database (released January 2016, 20121 entries) using the Proteome Discoverer (version 1.3, Thermo Fisher, Waltham, MA, USA). The maximum missed cleavages was set to 2, and trypsin was selected for protein digestion. The oxidation of methionines and protein N-terminal acetylation were defined as the variable modification, and the carbamidomethylation of cysteines was the fixed modification. The precursor mass tolerance was set to 15 ppm. Both maximum peptide and protein false discovery rates (FDR) were limited to 1%. FDR was calculated using a global target-decoy approach. The criteria used for protein identification was: two unique peptides, *p* < 0.05, and a false discovery rate (FDR) of less than 1% at the protein level. The significant differentially expressed proteins (DEPs) ([ALF group vs. ALF+MHLQD High group] and [ACLF group vs. ACLF+MHLQD High group]) were identified by [fold change > 2.0 or <0.5] and *p* < 0.05.

### 2.14. Bioinformatic Analysis

The DEPs were integrated to establish a protein-protein interaction (PPI) network using STRING, an online protein-protein interaction networks functional enrichment analysis tool (Version: 11.5) [21]. The minimum required interaction score was set at medium confidence (0.400). Active interaction source analysis was based on test-mining, experiments, databases, co-expression, neighborhood, gene fusion, and co-occurrence. The obtained PPI network was loaded into the software Cytoscape (an open-source software platform for visualizing complex networks, v3.7.1). Based on connectivity degree, the key nodes were analyzed using the Cytohubba (a connectivity score tool) plug-in in Cytoscape. Top 10% DEPs were ranked by the Monte Carlo Collisions (MCC) algorithm. Functional annotation and enrichment analysis of top 10% DEPs were performed using DAVID (the Database for Annotation, Visualization, and Integrated Discovery) Bioinformatics Resources [22], an online web tool. The Kyoto Encyclopedia of Genes and Genomes (KEGG) pathway enrichment analysis was performed on top 10% DEPs using the DAVID database. The enriched TOP 10 KEGG pathways were recorded. The data were visualized by Bioinformatics, an online web bioinformatics visualization tool, based on the confidence level (*p*-value), degree of enrichment, and the count value in each KEGG pathway.

### 2.15. Statistical Analysis

The data were expressed as means ± SD. The survival rate was analyzed using the Kaplan-Meier method. Differences between groups were compared by a one-way analysis of variance (ANOVA). If a statistically significant change was found (*p* < 0.05), a post hoc comparison was performed using Fisher’s LSD test.

## 3. Results

### 3.1. The Chemical Compositions of MHLQD Identified Using UPLC-Q-TOF-MS/MS

UPLC-Q-TOF-MS/MS detected a total of 65 chemical constituents in MHLQD aqueous extracts in terms of chromatography, elution behaviors, chemical composition, mass fragment patterns, and mass spectral library. Representative components of MHLQD were as follows: Ephedrine, Pseudoephedrine, Notopterol, Liquiritigenin, Anisic aldehyde, Formononetin, Forsythoside A, Amygdalin, and Forsythoside E. These chemical constituents were derived from the herbs in MHLQD, including *Ephedra alata* Decne., *Forsythia giraldiana* Lingelsh., *Prunus armeniaca* L., and *Glycyrrhiza aspera* Pall. (Figure S1, Table S1).

### 3.2. MHLQD Improved Survival Rates of ACLF and ALF Mice

**Figure 1:**
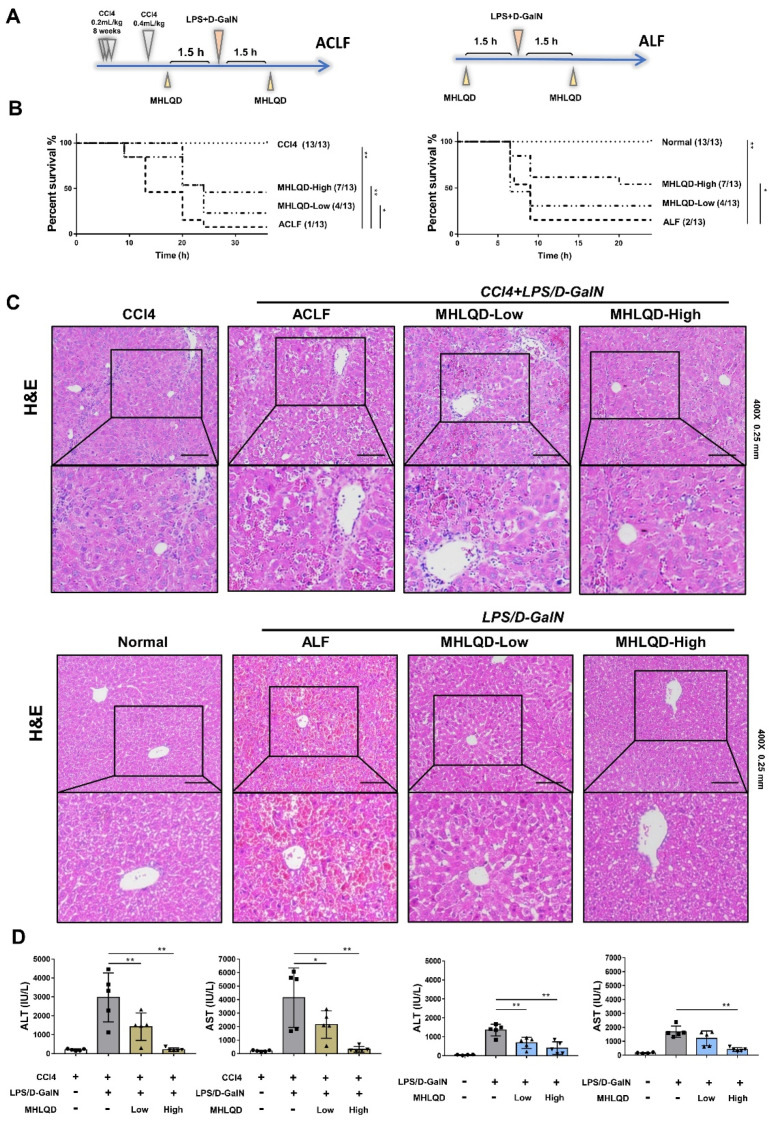
MHLQD improved survival rates and alleviated liver injury in ACLF and ALF mice. (**A**): The experimental design. The mice were injected with D−GalN (320 mg/kg) and LPS (50 ug/kg in survival experiments and 30 ug/kg in non-survival experiments) to establish the ALF model. The ACLF model was established through additional pretreatment with CCl_4_ for 8 weeks. MHLQD (40.5 or 81 g/kg) or the same volume (0.2 mL) of PBS was orally administrated to mice 1.5 h before and 1.5 h after LPS/D−GalN injection. (**B**): The survival experiment of ACLF and ALF mice (n = 13). (**C**): The H&E staining of ACLF and ALF mice liver. Scale bar: 0.25 mm. (**D**): Liver function levels (ALT and AST) in ACLF and ALF mice (n = 5). Data were presented as mean ± SD, * *p* < 0.05, ** *p* < 0.01. Abbreviation: MHLQD: MaHuang-LianQiao-ChiXiaoDou decoction, ACLF: acute-on-chronic liver failure, ALF: acute hepatic failure.

Using the ALF and ACLF model, we first observed a protective effect of MHLQD on mouse survival. In the control group, mice with ACLF and ALF eventually died after 10–36 h. We defined the dose of MHLQD via the human-to-mouse body drug dose conversion factor, and the usage of MHLQD was shown in Figure 1A. We found that both the high dose (81 g/kg) and low dose (40.5 g/kg) of MHLQD significantly improved survival rates at 36/24 h in ALF and ACLF mice, respectively (Figure 1B). These results implied that MHLQD is an effective treatment to protect the mice against ACLF and ALF.

### 3.3. MHLQD Alleviated Liver Injury in ACLF and ALF Mice

Histologically, lots of nuclear-free hepatocytes and infiltrated blood cells were observed in the liver of ACLF/ALF mice. Hepatocyte death and infiltrated blood cells were reduced in the MHLQD group (Figure 1C). Additionally, the liver surface of mice in the MHLQD group showed less extravasated blood compared to that of mice in the ALF/ACLF group (Figure S2). Compared with the mice in the ALF/ACLF group, the serum of MHLQD-treated mice displayed a shallower yellow color, which also implied that MHLQD improved intrahepatic blood clots. Moreover, MHLQD treatment significantly reduced serum alanine aminotransferase (ALT) and aspartate aminotransferase (AST) levels, compared with the ACLF and the ALF group (Figure 1D). These results further confirmed the protective effects of MHLQD on ACLF and ALF mice.

### 3.4. MHLQD Inhibited Inflammatory Responses in ACLF and ALF Mice

**Figure 2:**
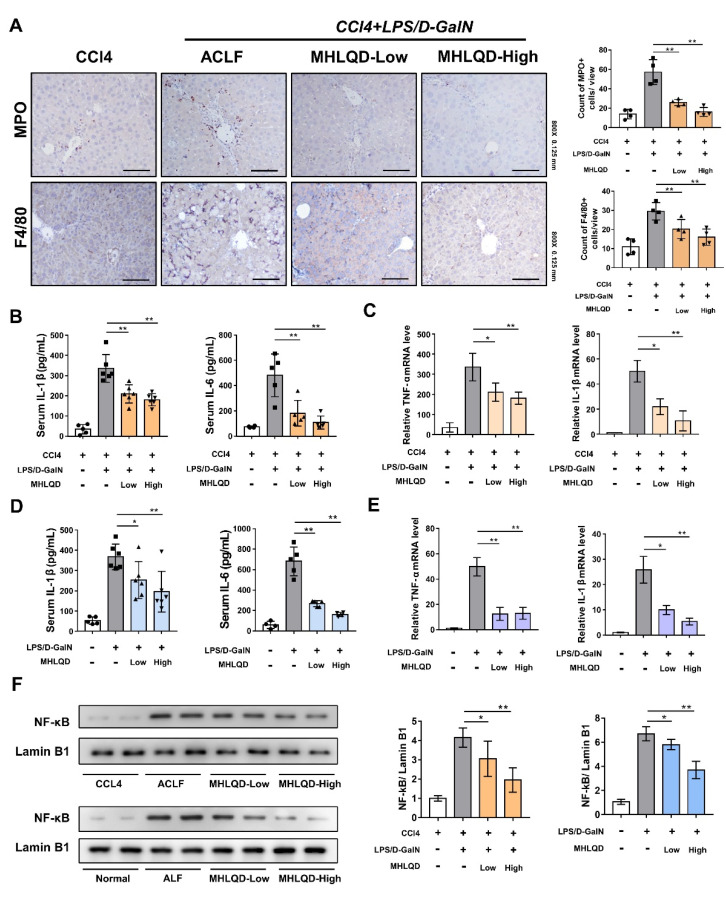
MHLQD alleviated liver injury and inflammatory response in ACLF and ALF mice. The mice were intraperitoneally injected with 320 mg/kg D−GalN and 30 ug/kg LPS to establish ACLF and ALF models. MHLQD (40.5 or 81 g/kg) or the same volume (0.2 mL) of PBS was orally administrated to mice 1.5 h before and 1.5 h after LPS/D−GalN injection. (A): The immunohistochemical staining of MPO and F4/80 in liver tissues of ACLF mice. Scale bar: 0.125 mm. (**B**): The serum levels of IL-1β and IL-6 in ACLF mice (n = 5). Results were shown as cytokine levels per milliliter of mice serum. (**C**): The relative mRNA expression levels of TNF-α and IL-1β in liver tissues of ACLF mice (n = 4). (**D**): The serum levels of IL-1β and IL-6 in ALF mice (n = 5). (**E**): The relative mRNA expression levels of TNF-α and IL-1β in liver tissues of ALF mice (n = 4). (**F**): The nucleus NF-kB expression in the liver tissues of ACLF and ALF mice. * *p* < 0.05, ** *p* < 0.01. Abbreviation: MHLQD: MaHuang-LianQiao-ChiXiaoDou decoction, ACLF: acute-on-chronic liver failure, ALF: acute hepatic failure.

Immunohistochemical (IHC) staining showed that MPO (a typical marker of neutrophil)-positive cells and F4/80 (a marker of monocyte/macrophage and kupffer cell)-positive cells in the ACLF and ALF groups increased. The MHLQD group exhibited fewer MPO-positive cells and F4/80-positive cells (Figure 2A and Figure S2). Cytokine levels indicated the severity of the systemic inflammatory responses. ELISA analysis showed that serum IL-1β and IL-6 were significantly lower in the MHLQD group compared with the ACLF or ALF groups (Figure 2B,D). PCR analysis showed that the expression of IL-1β and TNF-α in liver tissues of the MHLQD group was significantly reduced compared with the ACLF or ALF groups (Figure 2C,E). Moreover, MHLQD inhibited the nuclear transfer of NF-kB (Figure 2F). These results indicated that MHLQD reduced both local and systemic inflammation responses in ACLF and ALF mice.

### 3.5. MHLQD Reduced Hepatocyte Death in ACLF and ALF Mice

**Figure 3:**
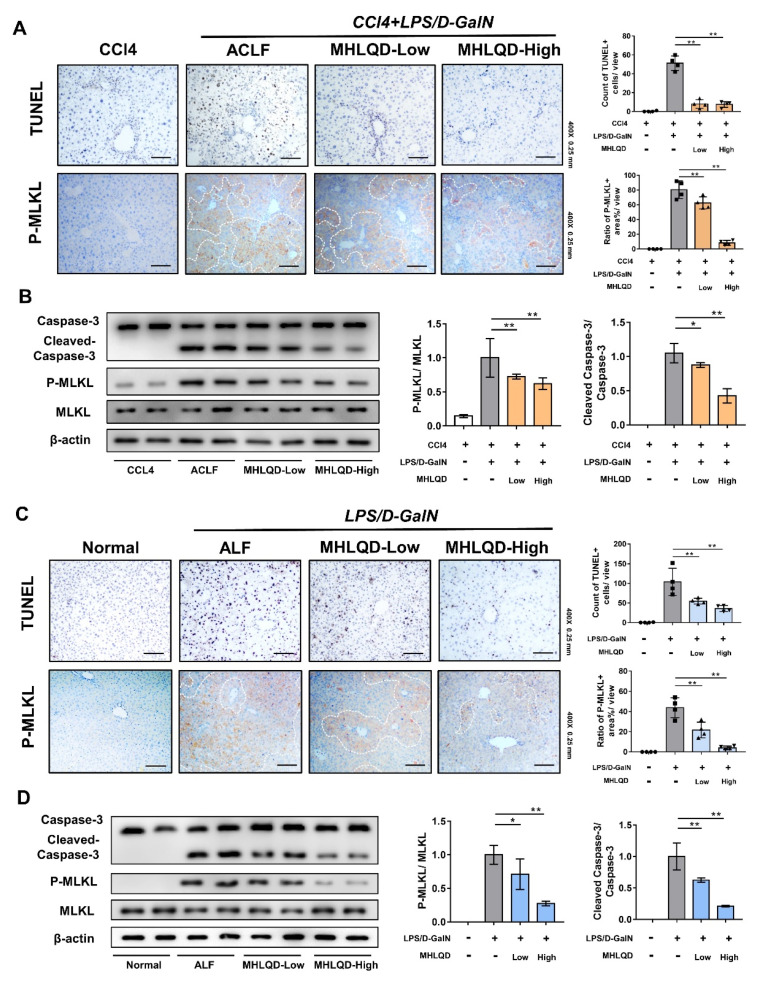
MHLQD suppressed hepatocyte necroptosis and apoptosis in ACLF and ALF mice. The mice were intraperitoneally injected with 320 mg/kg D−GalN and 30 ug/kg LPS to establish ACLF and ALF models. MHLQD or the same volume of PBS was orally administrated to mice 1.5 h before and 1.5 h after LPS/D−GalN injection. (**A**): The TUNEL staining and p-MLKL staining in liver tissues of ACLF mice. Scale bar: 0.25 mm. (**B**): The protein expression of Caspase-3 and p-MLKL/MLKL in liver tissues of ACLF mice. (**C**): The TUNEL staining and p-MLKL staining in liver tissues of ALF mice. Scale bar: 0.25 mm. (**D**): The protein expression of Caspase-3 and p-MLKL/MLKL in liver tissues of ALF mice. * *p* < 0.05, ** *p* < 0.01. Abbreviation: MHLQD: MaHuang-LianQiao-ChiXiaoDou decoction, ACLF: acute-on-chronic liver failure, ALF: acute hepatic failure.

TUNEL staining showed that TUNEL-positive hepatocytes in the ACLF/ALF group were higher than those in the CCl_4_/Normal group, respectively. MHLQD treatment significantly reduced the TUNEL-positive hepatocytes (Figure 3A,C). We further examined the expression of P-MLKL, which has been used as a necroptosis marker. The staining showed there were more P-MLKL-positive areas in the ACLF/ALF group than in the CCl_4_/Normal group, respectively. In contrast, the MHLQD group exhibited reduced P-MLKL-positive staining area, indicating a decrease in necroptosis hepatocytes. Moreover, we also detected the expression of cleaved-caspase-3 and p-MLKL, which are the molecular markers of apoptosis and necroptosis. The results also showed MHLQD significantly suppressed cleaved-caspase-3 and p-MLKL expression, compared with the ACLF and the ALF group (Figure 3B,D). Hepatocyte death induced by inflammatory cells-released inflammatory mediators is the direct cause of liver destruction [23,24]. Both apoptosis and necroptosis have been reported to be induced during ALF and ACLF [25,26]. These results showed that MHLQD could reduce hepatocyte death in ALF and ACLF mice.

### 3.6. MHLQD Reversed ACLF and ALF-Induced Platelet Activation

**Figure 4:**
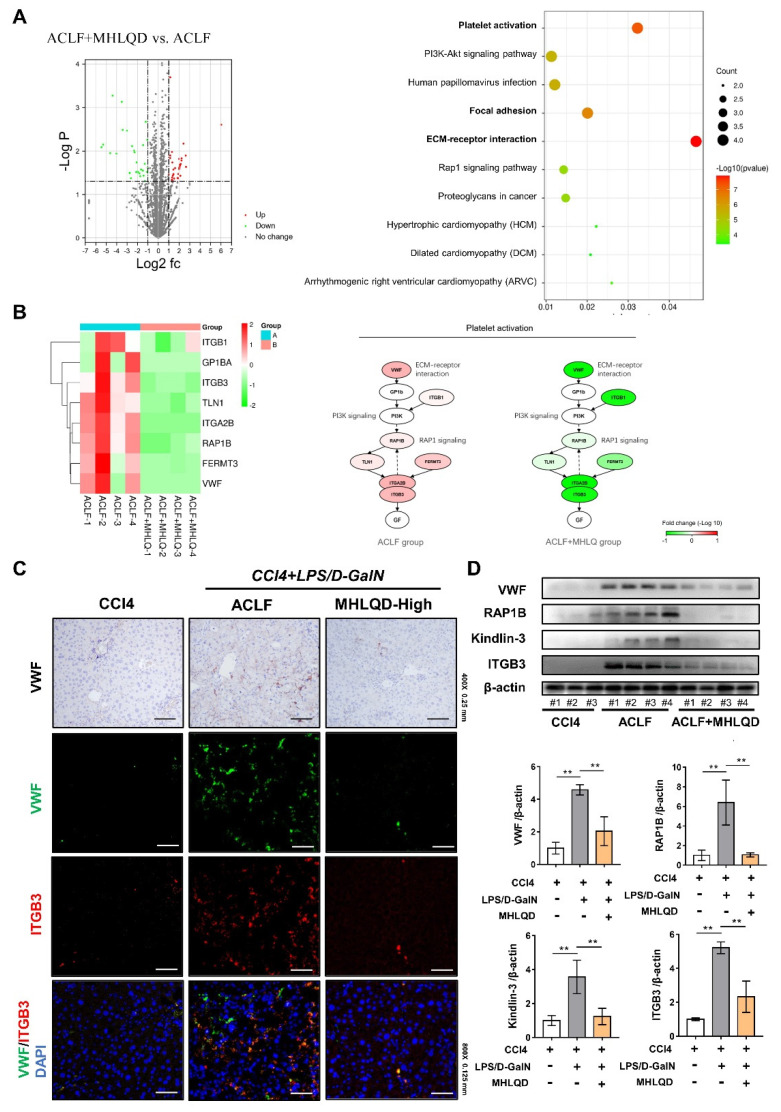
MHLQD inhibited VWF-induced platelet activation in ACLF mice. The mice were intraperitoneally injected with D−GalN and LPS to establish the ACLF model. MHLQD or PBS was orally administrated to mice 1.5 h before and 1.5 h after LPS/D−GalN injection. (**A**): KEGG enrichment analysis of top 10% differentially expressed proteins (DEPs) between the ACLF+MHLQD and the ACLF group. (**B**): The heatmap of protein expression and the signal transduction schema of platelet activation signaling between the ACLF+MHLQD and the ACLF group. (**C**): The immunohistochemical staining of VWF in liver tissues (Scale bar: 0.25 mm) and the colocalization of VWF (Green) and ITGB3 (Red) (Scale bar: 0.125 mm). (**D**): VWF-induced platelet activation signal was detected by western blot. ** *p* < 0.01. Abbreviation: MHLQD: MaHuang-LianQiao-ChiXiaoDou decoction, VWF: von Willebrand factor, ACLF: acute-on-chronic liver failure.

**Figure 5:**
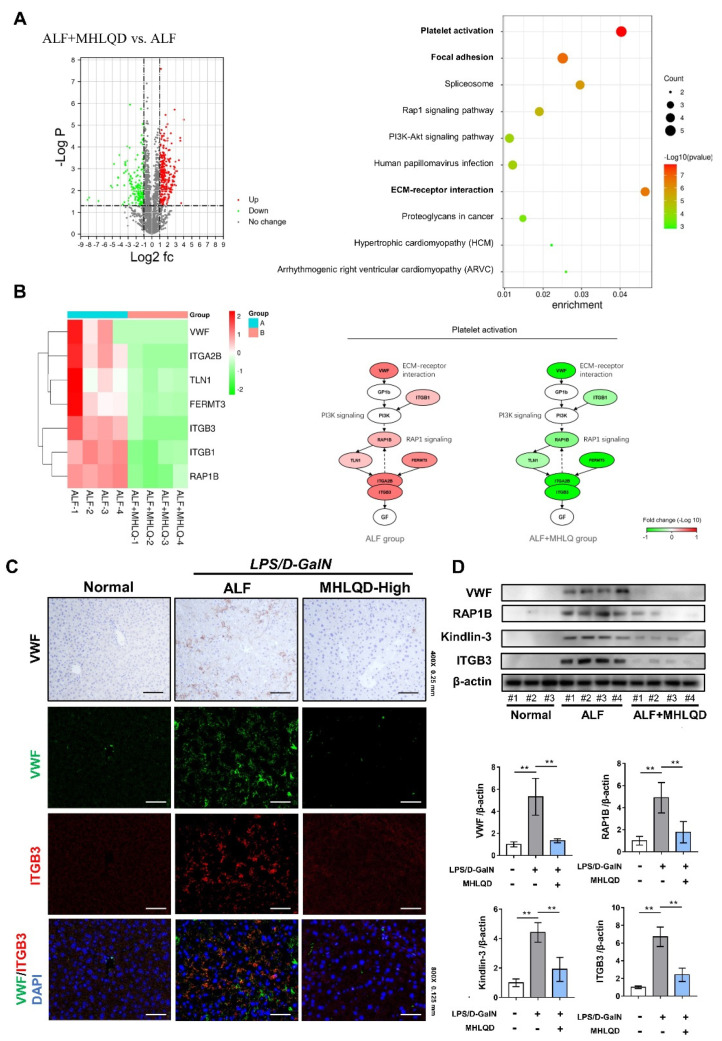
MHLQD inhibited VWF-induced platelet activation in ALF mice. The mice were intraperitoneally injected with D−GalN and LPS to establish the ALF model. MHLQD or PBS was orally administrated to mice 1.5 h before and 1.5 h after LPS/D−GalN injection. (**A**): KEGG enrichment analysis of top 10% DEPs between the ALF+MHLQD and the ALF group. (**B**): The heatmap of protein expression and the signal transduction schema of platelet activation signaling between the ALF+MHLQD and the ALF group. (**C**): The immunohistochemical staining of VWF in liver tissues (Scale bar: 0.25 mm) and the colocalization of VWF (Green) and ITGB3 (Red) (Scale bar: 0.125 mm). (**D**): VWF-induced platelet activation signal was detected by western blot. ** *p* < 0.01. Abbreviation: MHLQD: MaHuang-LianQiao-ChiXiaoDou decoction, VWF: von Willebrand factor, ALF: acute hepatic failure.

Although multiple mechanisms and targets are often involved in the effects of TCM formulas and natural herbs, studies have shown that herb formulas can target critical pathological processes and thus greatly improve disease progression [27,28]. In order to investigate the most important signaling pathway involved in the protective effects of MHLQD, proteomics analysis was used to explore the key mechanism of MHLQD in the treatment of ACLF/ALF. A total of 2680 proteins were detected by protein mass spectrometry. A total of 122 DEGs (57 down-regulated proteins and 65 up-regulated proteins) between ACLF and ACLF+MHLQD were identified. A total of 605 DEGs (257 down-regulated proteins and 348 up-regulated proteins) were identified between ALF and ALF+MHLQD. The top 10% of DEPs were shown in Figure S3. KEGG enrichment analysis showed that the optimized DEPs between ACLF and ACLF+MHLQD were highly enriched in platelet activation, focal adhesion, and ECM-receptor pathway (Figure 4A). Parallelly, the DEPs between ALF and ALF+MHLQD were highly enriched in ECM-receptor pathway, platelet activation, and focal adhesion (Figure 5A). Platelet activation is an important pathological process involving endothelial cell injury and disseminated intravascular coagulation (DIC), eventually leading to organ damage [29]. Because MHLQD protects both ALF and ACLF, and platelet activation is commonly altered in ALF and ACLF, platelet activation was selected for further analysis. Heatmap showed that MHLQD reduced the expression of VWF, TGB1, RAP1B, TLN1, FERMT3, ITGA2B, and ITGB3. VWF is a crucial trigger for inducing downstream signaling activation that ultimately results in platelet aggregation. Importantly, the platelet activation pathway involves signaling pathways, including the ECM-receptor pathway (VWF binds to GP1b) and RAP1 signaling (RAP1B/TLN1/ITGB3 signaling), both of which were significantly enriched in the DEGs of ALF/ALF+MHLQD and ACLF/ACLF+MHLQD (Figure 4B and Figure 5B). Therefore, we hypothesized that the VWF signaling might be the key mechanism of MHLQD.

### 3.7. MHLQD Inhibited VWF-Platelet String Generation

VWF-induced platelet activation forms VWF-platelet string to aggravate tissue damage. Intrahepatic VWF-platelet string is a characteristic of both ACLF and ALF. Aggregation of VWF-platelet string gives rise to intrahepatic blood clots, which may lead to inhibition of liver regeneration, formation of immune thrombus, and circulatory disturbance-induced tissue necrosis [30]. IHC exhibited high expression of VWF in the liver of ACLF and ALF groups, as well as the inhibition of VWF expression in the liver under MHLQD treatment. Tissue immunofluorescence of VWF and ITGB3 confirmed the highly expressed VWF-platelet string in the ALF and ACLF groups, which was inhibited in the MHLQD group (Figure 4C and Figure 5C). Consistently, the expression levels of VWF, Rap1B, Kindlin-3, and ITGB3 were elevated in the ALF and ACLF groups, and this result was reversed by MHLQD treatment (Figure 4D and Figure 5D). These results suggest that the activated VWF-platelet string pathways were involved in ALF and ACLF, which were inhibited by MHLQD.

### 3.8. Inhibition of VWF Intercepted the Hepatoprotective Effects of MHLQD

**Figure 6:**
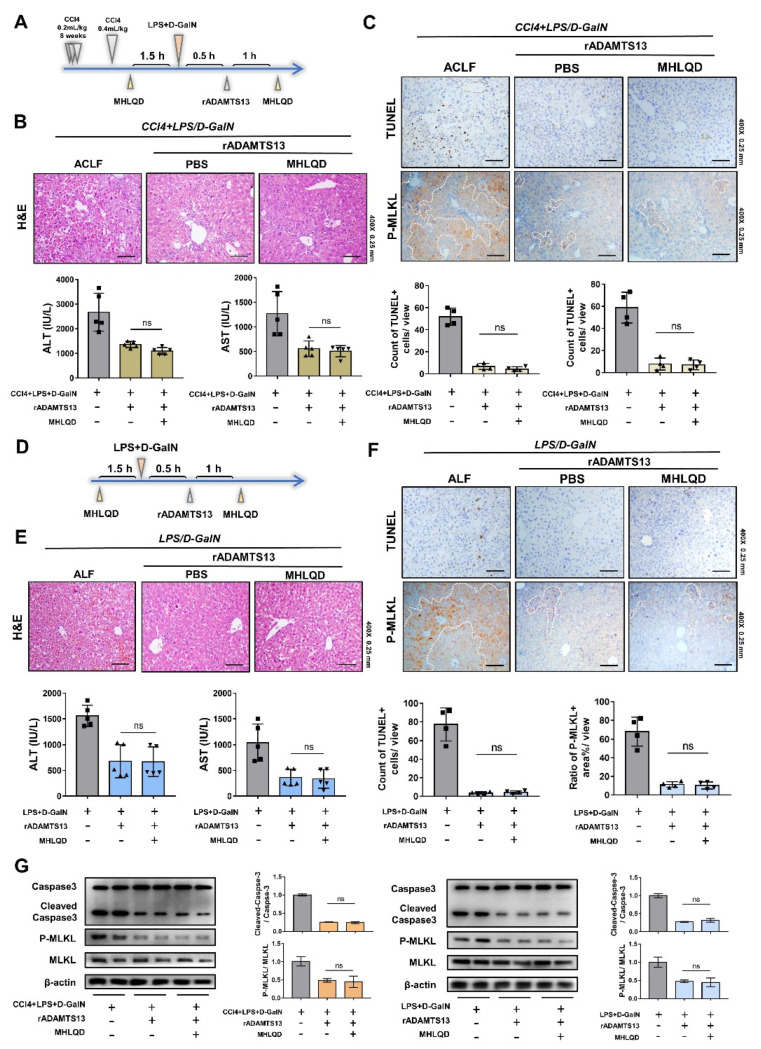
The therapeutic effect of MHLQD could not be replicated in VWF inhibitor-treated mice. The mice were intraperitoneally injected with D−GalN and LPS to establish ACLF and ALF models. The rADAMTS13 (200 ug/kg), a VWF clearer, was injected into mice 0.5 h after D−GalN/LPS treatment. MHLQD or PBS was orally administrated to mice 1.5 h before and 1.5 h after LPS/D−GalN injection. (**A**): The experimental design of ACLF mice. (**B**): The H&E staining of the liver and the liver function levels (ALT and AST) in ACLF mice (n = 5). Scale bar: 0.25 mm. (**C**): The TUNEL staining and p-MLKL staining in liver tissues of ACLF mice. Scale bar: 0.25 mm (**D**): The experimental design of ALF mice. (**E**): The H&E staining of the liver and the liver function levels (ALT and AST) in ALF mice (n = 5). Scale bar: 0.25 mm. (**F**): The TUNEL staining and p-MLKL staining in liver tissues of ALF mice. Scale bar: 0.25 mm. (**G**): The protein expression of Caspase 3 and P-MLKL/MLKL in liver tissues of ACLF and ALF mice. ns: no significant. Abbreviation: MHLQD: Ma-Huang-LianQiao-ChiXiaoDou decoction, VWF: von Willebrand factor, ACLF: acute-on-chronic liver failure, ALF: acute hepatic failure.

To determine whether the effect of MHLQD depended on the inhibition of VWF signaling, an accepted VWF inhibitor, rADAMTS13, was injected into the tail vein of mice. As expected, rADAMTS13 treatment downregulated liver function levels (ALT and AST) and alleviated histological changes in ACLF and ALF mice, indicating that VWF signaling activation was involved in liver injury blocked by rADAMTS13 (Figure 6B,E). However, MHLQD could not further reduce the serum ALT and AST in rADAMTS13-treated ACLF and ALF mice (Figure 6B,E). TUNEL and P-MLKL staining showed no difference between the MHLQD+rADAMTS13 group and the rADAMTS13 group (Figure 6C,F). Moreover, western blotting showed lower levels of cleaved-caspase3 and P-MLKL in rADAMTS13-treated mice (Figure 6G). MHLQD could not further reduce the activation of caspase3 and MLKL. MHLQD was unable to further alleviate liver injury and cell death in rADAMTS13-treated mice. Therefore, these results suggest that the protective effects of MHLQD are ultimately partly ascribed to the inhibition of VWF signaling activation.

### 3.9. Short-Term Treatment with MHLQD Showed No Toxicity

**Figure 7:**
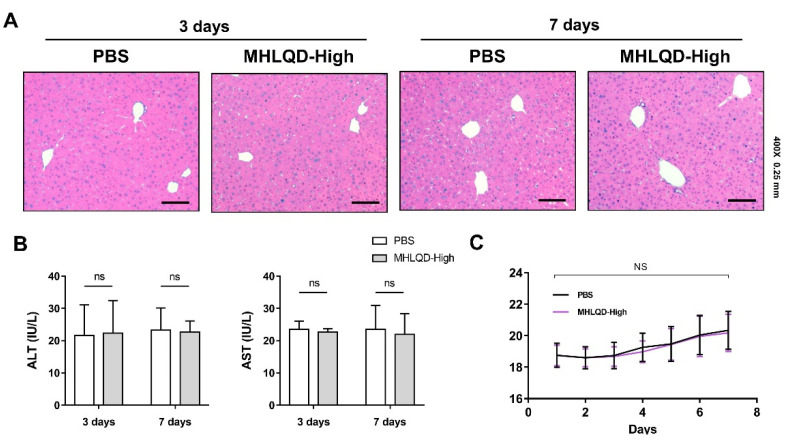
Short-term treatment of MHLQD did not show toxicity. The mice were randomly divided into (1) the control group, and (2) the MHLQD group. The mice were intragastrically administrated with MHLQD (81 g/kg) or the same volume (0.2 mL) of PBS twice a day. The mice were sacrificed on the third day or the seventh day. (**A**): The H&E staining of the liver section. Scale bar: 0.25 mm. (**B**): The liver function levels (ALT and AST) of the mice (n = 5). (**C**): The body weight curves of the mice (n = 5). Abbreviation: MHLQD: MaHuang-LianQiao-ChiXiaoDou decoction.

To observe whether MHLQD had potential toxicity, we tested the effect of MHLQD in a short-term administrate. Mice were intragastrically administrated with a high dose of MHLQD for 3 days or 7 days. Body weight curves showed no significant difference between MHLQD-treated mice and normal saline-treated mice (Figure 7A). Histological detection demonstrated that the MHLQD-treated mice showed normal morphology of hepatocytes, and there was no obvious thickening of hepatic sinuses, implying that MHLQD treatment for 3 days or 7 days did not cause pathological changes in the liver (Figure 7B). Moreover, MHLQD treatment did not affect the liver function levels, which remained within the normal range for both the MHLQD-treated mice and normal saline-treated mice (Figure 7C).

## 4. Discussion

ACLF and ALF are serious liver diseases with short-term mortality rates of 30–50% [31]. In this study, we investigated the protective effect and the potential mechanism of MHLQD in ACLF and ALF mice models. Results showed that MHLQD improved survival in ACLF and ALF mice, and alleviated liver damage and histological changes. Proteomic analysis showed that platelet activation induced by the VWF signaling might be the main target of MHLQD. Using a VWF signaling inhibitor, we confirmed that the protective effect of MHLQD partly depended on inhibiting VWF signaling-induced platelet activation. Our results provided evidence that MHLQD could be an effective therapeutic strategy for ACLF and ALF patients. 

Pathogen infection is a main precipitating event in ACLF and ALF inducement and decompensation [32]. LPS is an antigen from gram-negative bacteria which could highly activate the innate immune system. During liver injury, gut leakage leads to excessive LPS entering the portal vein circulation to promote the release of pro-inflammatory cytokines [33]. D−GalN is a naturally occurring amino sugar in the host that can deplete UTP to suppress the synthesis of anti-cell death proteins in hepatocytes [34]. Since rodents are resistant to LPS, both D−GalN and LPS were used to establish an inflammatory factor-dominated hepatic injury model [35] to mimic the pathology of acute hepatitis. The CCl_4_+LPS/D−GalN-induced ACLF mouse is a well-established preclinical model which has been widely adopted by investigators recently [36,37,38]. The liver damage in this model was easy to control via different doses of CCl_4_+LPS/D−GalN. Therefore, the CCl_4_+LPS/D−GalN-induced ACLF mouse model has also been used to test the potential therapeutic effects of drugs on ACLF [39]. We replicated murine models of ACLF and ALF with mortality by LPS+D−GalN and CCl_4_, as previously described [36,40]. Compared with ALF mice, latent cirrhotic ACLF mice showed a partially differentiated phenotype, including more serious liver function damage (higher ALT and AST), delayed time to death, and different histological morphology. We observed more severe necroptosis hepatocytes in ACLF mice compared with ALF mice. These ACLF and ALF models were consistent with most of the clinical features and pathogenesis of ACLF/ALF.

During ALF and ACLF, many hepatocytes underwent death, releasing DAMP to trigger inflammation, and the excessive innate responses further induced hepatocyte death and systemic immune response syndrome [23]. We detected high expression of IL-6, IL-1β, and TNF-α, as well as infiltrated innate immune cells (macrophages and neutrophils) in the liver of ALF and ACLF mice. MHLQD treatment significantly inhibited the inflammatory cytokines and infiltration of immune cells. 

Fulminant hepatocyte death was identified as the pivotal pathological mechanism of liver failure. Apoptosis and necroptosis are considered two typical modes of cell death in acute liver disease [27,28,41,42], both of which are triggered by the tumor necrosis factor receptor superfamily. Apoptotic signaling via mitochondrial-dependent or -independent caspase cascade reaction is culminated by caspase-3 activation. Necroptosis involves necrosome assembly and phosphorylation of mixed lineage kinase domain-like protein (MLKL) [43]. Apoptosis and necroptosis are recognized as alternative outcomes of the same initiating factors [44]. However, apoptosis and necroptosis frequently coexist during the disease caused by the considerable crosstalk in pathways, providing another backup mechanism for cell death failure [45]. In our study, ALF/ACLF mice showed high rates of TUNEL-positive hepatocytes and p-MLKL-positive staining areas in the liver. High expression of Cleaved-Caspase-3 and p-MLKL by western blotting implied serious hepatocyte apoptosis and necroptosis, which was effectively suppressed by MHLQD. It is worth mentioning that the hepatocyte cytoskeleton of ACLF mice was more complete than that of ALF mice. Consistent with a recent report [46], our results suggested that chronic liver injury induced a differentiated proportion between apoptosis and necroptosis.

Due to the multiple components of TCM formulas, mechanism analysis of the herb formula is often superficial and one-sided. The therapeutic effects of TCM formula have been considered to be mediated by dominant and combined effects [19,20]. Recently, proteomics technology has been applied to comprehensively analyze the dominant effects of TCM formula [18]. In this study, we used high-throughput screening to explore the mechanism of MHLQD in ACLF and ALF by proteomics. Proteomic analysis indicated that VWF signaling and VWF-induced platelet activation might be the important mechanisms of MHLQD treatment in ACLF and ALF. These therapeutic effects were confirmed by histological staining and western blotting. Further, we applied rADAMTS13, a VWF cleaner, to investigate whether the therapeutic effect of MHLQD was mainly dependent on VWF-induced platelet activation. Results showed that rADAMTS13 suppressed VWF and platelet in the liver, and ameliorated liver injury. Importantly, no distinct differences were detected in the histology of liver injury, hepatocyte death, and immunoreactivity between the MHLQD+rADAMTS13 group and the rADAMTS13 group, implying that the therapeutic effect of MHLQD could not be replicated in rADAMTS13-treated mice. In conclusion, based on these results, inhibition of the VWF-mediated platelet reaction might be the key mechanism of the therapeutic effect of MHLQD in ACLF/ALF.

VWF is an adhesion protein that can form the microthrombus to promote liver damage. A recent study revealed that VWF was independently associated with a decreased risk of further decompensation, ACLF, and death. Decreasing VWF levels in patients indicated a lower risk and better outcome [47]. Previous research revealed that increased VWF could recruit platelets to induce intrahepatic hypercoagulation status and develop the formation of microthrombus [14,48]. Intrahepatic microthrombogenesis could induce ischemia and hypoxia in local tissue, hepatocyte death, thrombotic immune response, and liver regeneration disorder [17]. VWF could bind to platelets to generate platelet-VWF string, which was correlated with disease severity in experimental animal models [32]. Our results showed that platelet-VWF string (the co-expression of VWF and ITGB3) was detected to be diffusely distributed in the diseased liver. MHLQD significantly inhibited the formation of platelet-VWF string. We suggested that MHLQD could suppress VWF expression, which was insufficient to recruit platelets. Recent studies have begun to focus on the role of VWF in liver failure. The mechanisms controlling VWF remain obscure. Some studies suggested that activation or damage of endotheliocytes is a possible mechanism of VWF leakage [32]. On the other hand, platelet activation not only produces thrombose but also accelerates the inflammatory reaction. Platelet-derived factors, including lipids, purines, nucleic acids, and cytokines, drive inflammasomes of macrophages and neutrophils to accelerate the inflammatory response. Ras-related protein 1 (rap1) is a key convergence point for multiple-platelet signaling, leading to the activation of integrin αIIbβ3 (including ITGB3 and ITGA2B subunits). Rap1B is the major component of total platelet rap1 proteins in murine platelet [49]. Kindlin-3 assists the downstream signal conduction of rap1B to support the activation of integrin αIIbβ3 and platelet aggregation [50]. We detected that elevated expression of rap1B, Kindlin-3, and ITGB3 in ALF/ACLF mice, and MHLQD inhibited this signaling.

We observed that VWF clearance could completely block platelets and alleviate hepatocyte death in ACLF and ALF mice. Studies have shown that VWF knockout, or VWF depletion, reduced cell death in murine models [32,51,52]. VWF synthesis was restricted to megakaryocytes and endothelial cells [17]. However, the source of VWF during liver failure remains unclear. It was thought that VWF and platelet-induced microthrombus formation caused circulation disorder and promoted hepatocyte death [16]. On the other hand, recent studies have reported that VWF could mediate the recruitment of monocytes, neutrophils, and T-cells [53]. The crosstalk between platelets and immune cells might result in hepatocyte death. In reality, patients with liver failure showed a state of intrahepatic hypercoagulable and extrahepatic coagulation failure [17]. Clinical use of all antithrombotic therapies was limited due to the risk of bleeding. VWF is a potent recruiter of platelet in ALF/ACLF, which drives platelet translocation. Therefore, anti-VWF, instead of anti-platelet, would be a more effective therapeutic strategy. We found that MHLQD could target VWF to suppress platelet activation. Thus, MHLQD could be used as a VWF inhibitor to induce antithrombotic effects. We will aim to investigate the release mechanism of VWF in follow-up studies.

MHLQD is an ancient herb prescription. The drug dose we adopted was based on the ancient document *Treatise on Febrile Diseases Caused by Cold*, which was in line with the current clinically acceptable range [54,55]. In the present study, we established a highly lethal murine model. In reality, low doses of MHLQD have acceptable effects. In clinical application, combined with existing supportive treatment, we suggest that the dose of MHLQD could be reduced.

Given the possible risk of herbal-induced liver damage, the side effects of herb preparation could not be ignored. MHLQD is constituted of eight herbs, and more than 40% of the herbs of MHLQD are food source plants such as ginger, Phaseolus calcaratus, and jujube. Our short-term toxicity test showed no apparent toxicity at the high dose of MHLQD. In the present study, to avoid possible changes to the chronic inflammatory state in mice with chronic liver disease induced by long-term administration of MHLQD, we only administrated MHLQD twice, 1.5 h before and 1.5 h after LPS/D−GalN injection, to prevent inflammatory cytokine storm. In our further study, we will investigate the pharmacological effect and safety of long-term administration of MHLQD in mice with chronic liver disease, and whether it can improve immune status and liver microenvironment in mice with chronic liver disease to avoid the liver microenvironment-induced inflammation amplification effect during acute inflammation.

In the present study, we found that MHLQD could ameliorate liver injury, alleviate inflammatory response, and inhibit hepatocyte death in the experimental ACLF and ALF murine models. Concerning the mechanism of action of MHLQD, proteomic analysis was used, and the results showed that platelet activation induced by VWF signaling might be involved in the protective effect of MHLQD.

## 5. Conclusions

Our results suggest that MHLQD is a potential therapy for ACLF and ALF. MHLQD could protect hepatocytes from death during liver failure. The effect of MHLQD ultimately depended in part on the inhibition of VWF-induced platelet activation.

## Figures and Tables

**Table 1:** The ingredients of the herbal formula MHLQD.

English Name	Latin Name	Part Used	Dosage
Ephedra	*Ephedra alata* Decne.	Stem	5.4 g/kg
Forsythia	*Forsythia giraldiana* Lingelsh.	Seed	5.4 g/kg
Bitter Apricot Seed	*Prunus armeniaca* L.	Seed	2.7 g/kg
Phaseolus Calcaratus	*Phaseolus acutifolius* A.Gray	Seed	5.4 g/kg
Jujube	*Ziziphus jujuba* Mill.	Mature Fruits	5.4 g/kg
Catalpa White	*Catalpa ovata* G.Don	Velamen	5.4 g/kg
Ginger	*Zingiber officinale* Roscoe	Rhizome	5.4 g/kg
Glycyrrhiza	*Glycyrrhiza aspera* Pall.	Root, rhizome	5.4 g/kg

## Data Availability

The datasets used and/or analyzed during the current study are available from the corresponding author (Kong, X., xiaonikong@shutcm.edu.cn) on reasonable request.

## Supplementary Materials

The following supporting information can be downloaded at: https://www.mdpi.com/article/10.3390/cells11213368/s1, Figure S1: Total ion flow chromatogram by UPLC/Q-TOF-MS/MS; Figure S2: MHLQD alleviates liver injury in ACLF and ALF mice; Figure S3: The network targets from top 10% DEPs between ALF/ACLF+MHLQD and ALF/ACLF group. Table S1: The chemical compositions of MHLQD identified by UPLC-Q-TOF-MS/MS.
