# Supplementary material for: Ancient Herbal Formula Mahuang Lianqiao Chixiaodou Decoction Protects Acute and Acute-on-Chronic Liver Failure via Inhibiting von Willebrand Factor Signaling
Source: Cells. 2022 Oct 25;11(21):3368. doi: 10.3390/cells11213368 (PMC9656135; doi:10.3390/cells11213368)
Supplement: Supplementary file 1 [file cells-11-03368-s001.zip › Supplementary materials/Table S1.pdf]

Table S1. The profile determined of MHLQD by UPLC-MS (Top 50)

Name	Formula	MW	RT (pos)	RT (neg)	Area (Max.)
Ephedrine	C10 H15 N O	165.11552	5.12		79451562.26
Pseudoephedrine	C10 H15 N O	165.11552	5.38		65640047.17
18 β-Glycyrrhetintic acid	C30 H46 O4	470.34021	13.882		26413379.82
Notopterol	C21 H22 O5	322.12121	10.778		8188832.987
Liquiritigenin	C15 H12 O4	256.07405	8.663		8009541.742
Skimmin	C15 H16 O8	324.08523	8.507		7210497.795
7-Hydroxycoumarin	C9 H6 O3	162.03205	8.512		7158119.715
2-Pyrrolidinecarboxylic acid	C5 H9 N O2	115.06337	0.955		6065416.838
Anisic aldehyde	C8 H8 O2	136.05267	2.819		5730705.606
L-Leucine	C6 H13 N O2	131.09496	1.745		5687952.499
Quillaic acid	C30 H46 O5	486.33518	12.543		5045576.714
L-Phenylalanine	C9 H11 N O2	148.05275	3.805		4656926.41
Bisdemethoxycurcumin	C19 H16 O4	308.10538	9.222		4055185.915
Forsythoside A	C29 H36 O15	624.20509		8.521	3193304.145
Formononetin	C16 H12 O4	268.07393	10.387		3155762.865
Vitexin rhamnoside	C27 H30 O14	578.16454	8.44		2825608.655
Diammonium glycyrrhizinate	C42 H62 O16	822.40272		13.888	2816112.898
Amygdalin	C20 H27 N O11	457.15892		6.886	2523399.354
Forsythoside E	C20 H30 O12	462.17436		6	2504913.709
Isoschaftoside	C26 H28 O14	282.07428	7.829		2379550.422
Morin	C15 H10 O7	302.04332	8.463		2326631.01
Adenosine	C10 H13 N5 O4	267.09749	1.906		2150546.526
Trigonelline HCL	C7 H7 N O2	137.04784	0.916		2062057.051
Ononin	C22 H22 O9	430.12682	10.387		2007785.292
Xanthoxyline	C10 H12 O4	196.07404	5.532		2001532.858
Caffeic acid	C9 H8 O4	180.04262	8.183		1929985.46
Dipotassium glycyrrhizinate	C42 H60 O16	822.40653	13.875		1704669.033
Loganic acid	C16 H24 O10	376.13734		5.511	1616711.111
(+)-Magnoflorine	C20 H23 N O4	341.16347	7.343		1510134.905
Hordenine	C10 H15 N O	165.11592	2.561		1483051.019
Ferulaldehyde	C10 H10 O3	178.06332	10.318		1415638.087
Alisol C	C32 H48 O6	528.34596	12.419		1319525.35
Ursonic acid	C30 H46 O3	472.35614	11.341		1290299.788
Isoliquiritin	C21 H22 O9	418.12697	10.287		1228684.841
Guanine	C5 H5 N5 O	134.0235	2.392		1056604.008
Cianidanol	C15 H14 O6	290.07985	6.605		1014161.627
Betaine	C5 H11 N O2	117.07893	0.911		1013171.486
Stachydrine	C7 H13 N O2	143.09494	0.963		1010692.89
Citric acid	C6 H8 O7	192.02792		0.991	928850.0926
Isorhamnetin	C16 H12 O7	316.05892	9.12		925833.326
Germacrone	C15 H22 O	218.16742	10.683		876772.8121
Vicenin II	C27 H30 O15	594.15953	7.314		865009.932
Hyperoside	C21 H20 O12	464.09642	8.683		835639.6841
2-Hydroxy-4-methoxybenzaldehyde	C8 H8 O3	152.04778	6.869		762971.7692
Glabrolide	C30 H44 O4	486.33525	11.391		739221.2948
Hydroxygenkwanin	C16 H12 O6	300.06404	9.665		623484.4753
Rutin	C27 H30 O16	610.15441	8.47		617236.3477
Adenine	C5 H5 N5	118.02824	1.882		581797.3941
Icaritin	C21 H20 O6	368.127	15.391		568449.4486
p-Coumaric acid	C9 H8 O3	164.04784	1.532		528500.7528
