# Supplementary figures and images for: Ancient Herbal Formula Mahuang Lianqiao Chixiaodou Decoction Protects Acute and Acute-on-Chronic Liver Failure via Inhibiting von Willebrand Factor Signaling
Source: Cells. 2022 Oct 25;11(21):3368. doi: 10.3390/cells11213368 (PMC9656135; doi:10.3390/cells11213368)

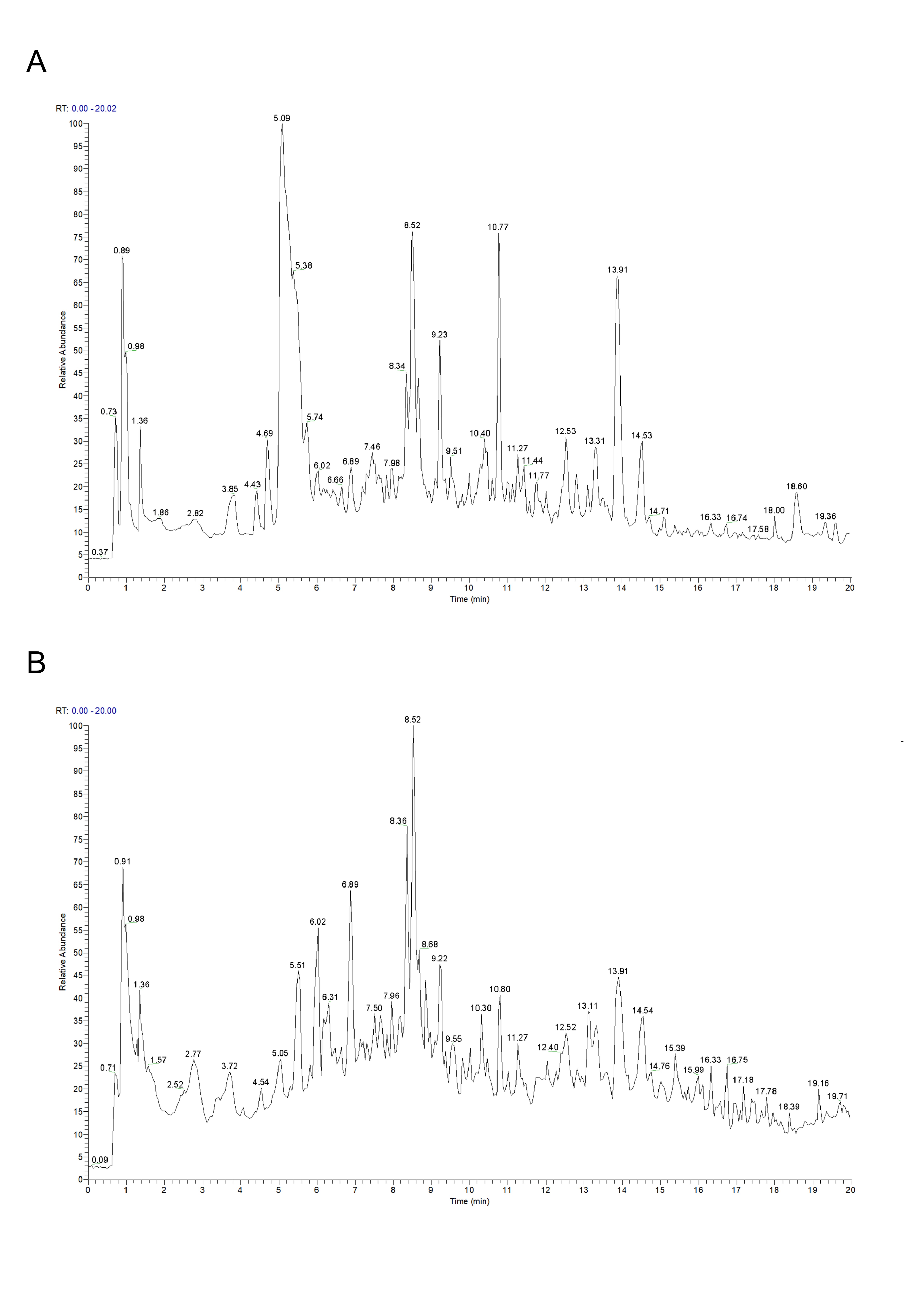

Supplement: Supplementary file 1 [file cells-11-03368-s001.zip › Supplementary materials/Figure S1.jpg]

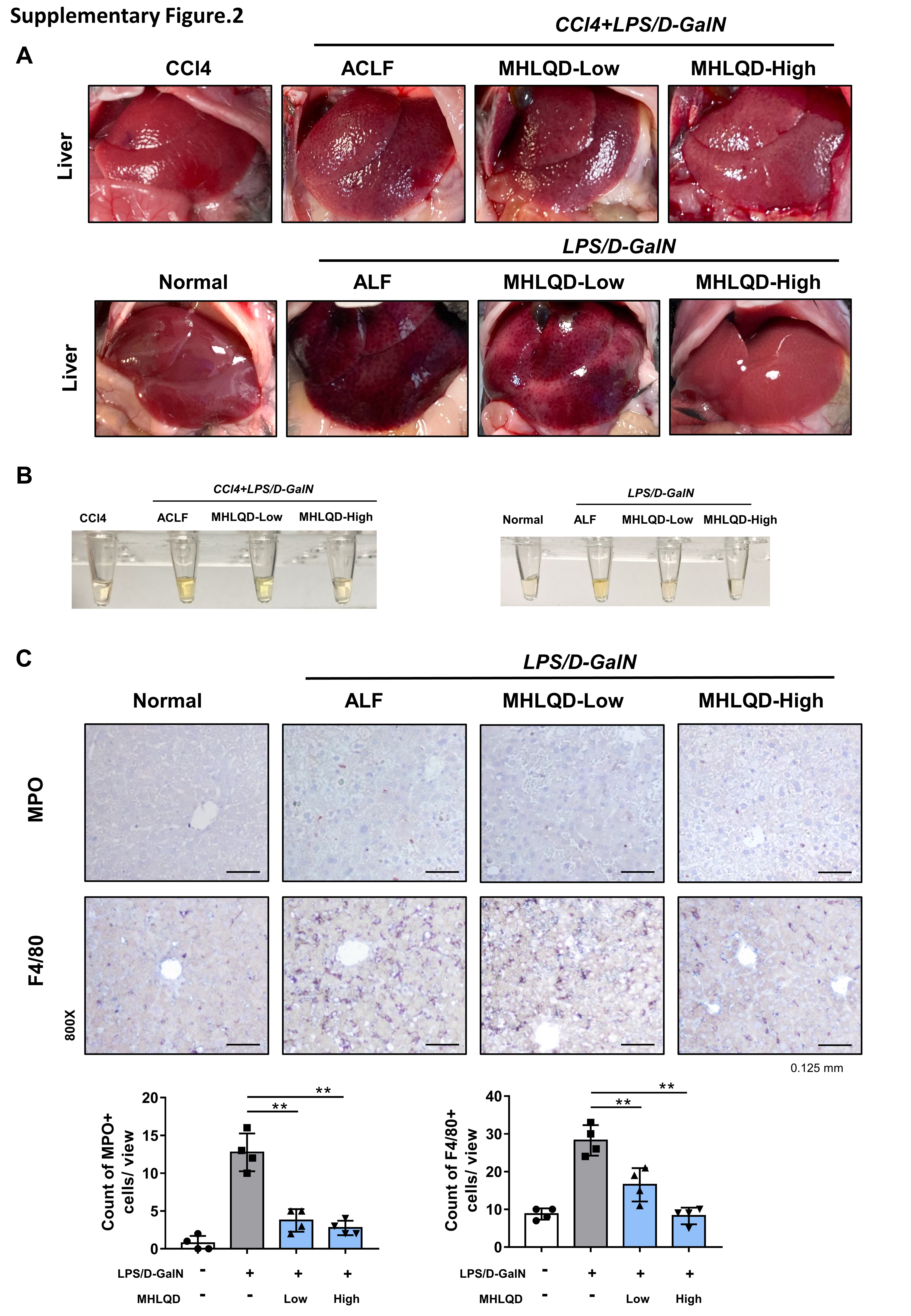

Supplement: Supplementary file 1 [file cells-11-03368-s001.zip › Supplementary materials/Figure S2.jpg]

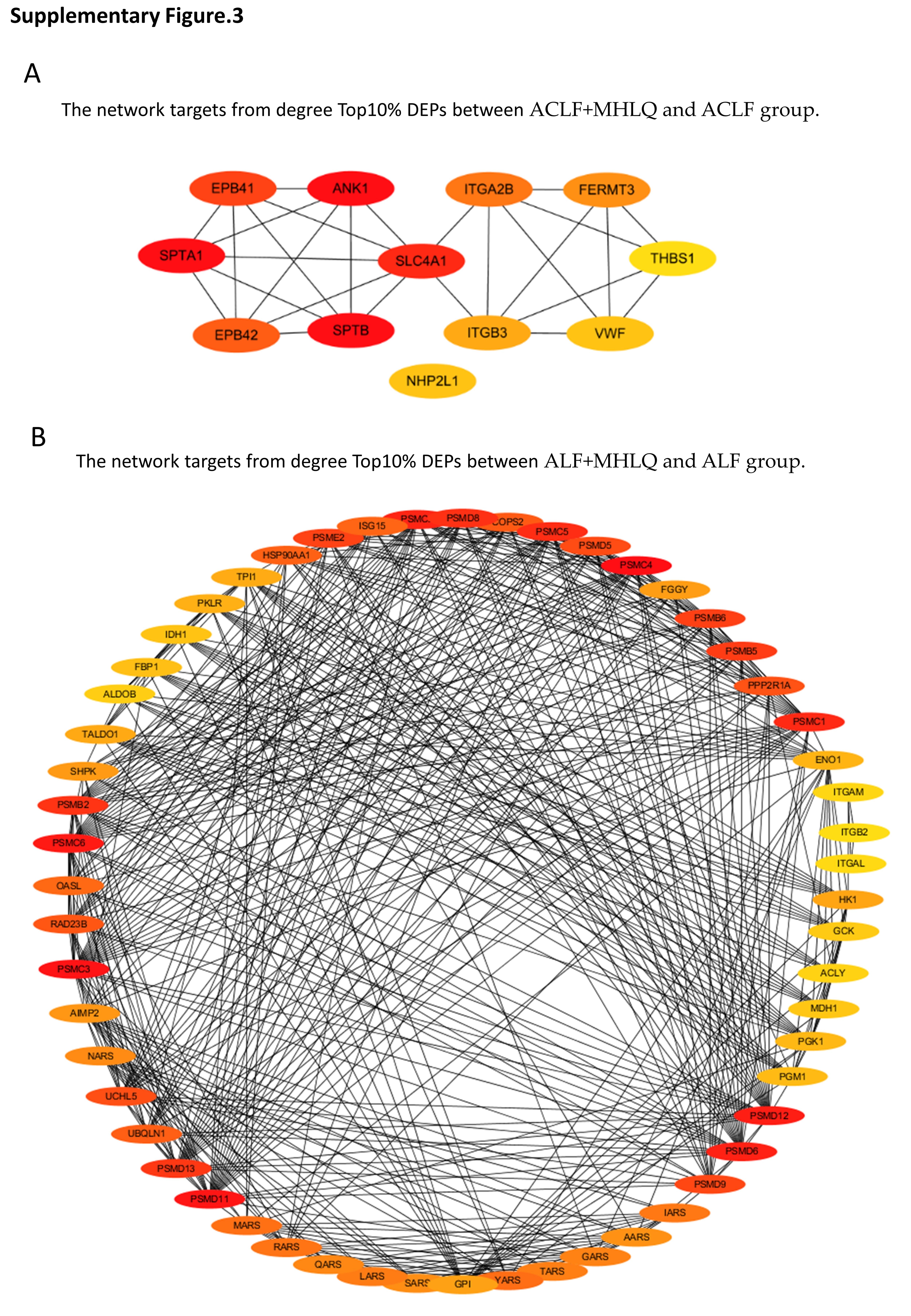

Supplement: Supplementary file 1 [file cells-11-03368-s001.zip › Supplementary materials/Figure S3.jpg]
